# Supplementary figures and images for: A modular protein language modelling approach to immunogenicity prediction
Source: PLoS Comput Biol. 2024 Nov 11;20(11):e1012511. doi: 10.1371/journal.pcbi.1012511 (PMC11581412; doi:10.1371/journal.pcbi.1012511)

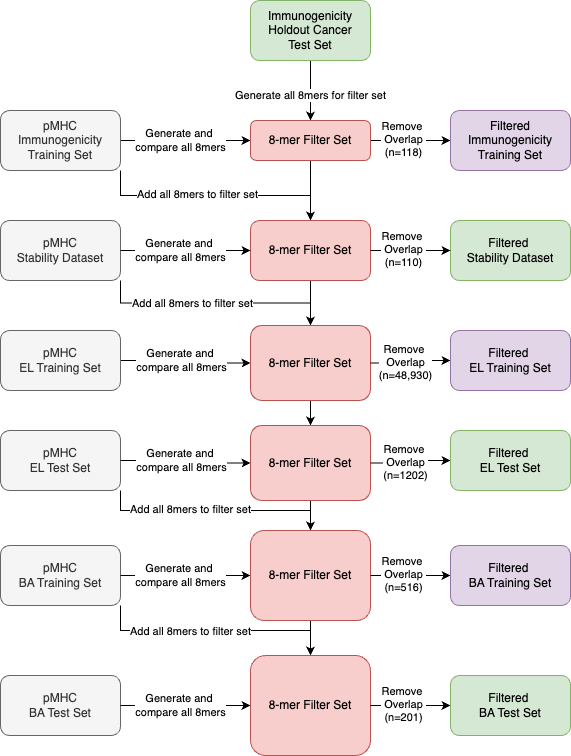

Supplement: S1 Fig — Filtering strategies for ensuring no data leakage between training steps, including for the sub-module tasks (A) and the cross-validation strategy ensuring all epitopes with matching 8mer substrings appear in the same fold. All data is filtered based on matches of 8 consecutive amino acids in the peptide. (TIFF) [file pcbi.1012511.s001.tiff]

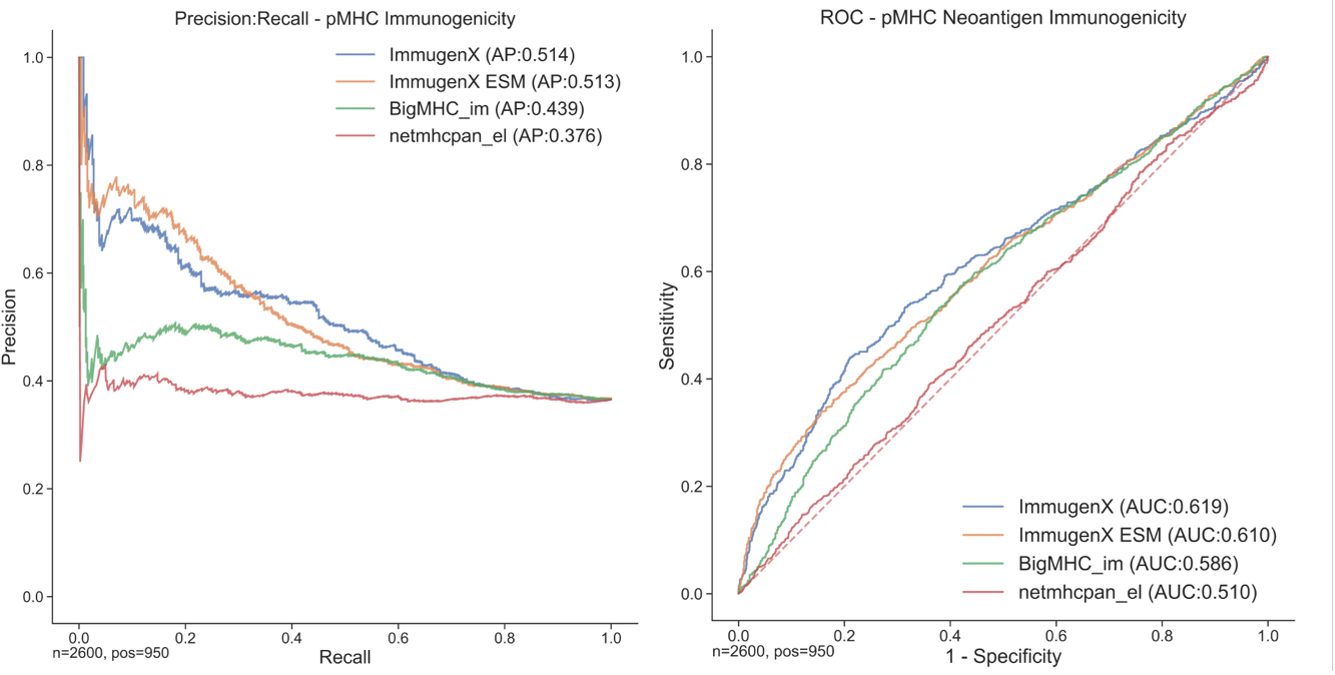

Supplement: S2 Fig — (TIFF) [file pcbi.1012511.s002.tiff]

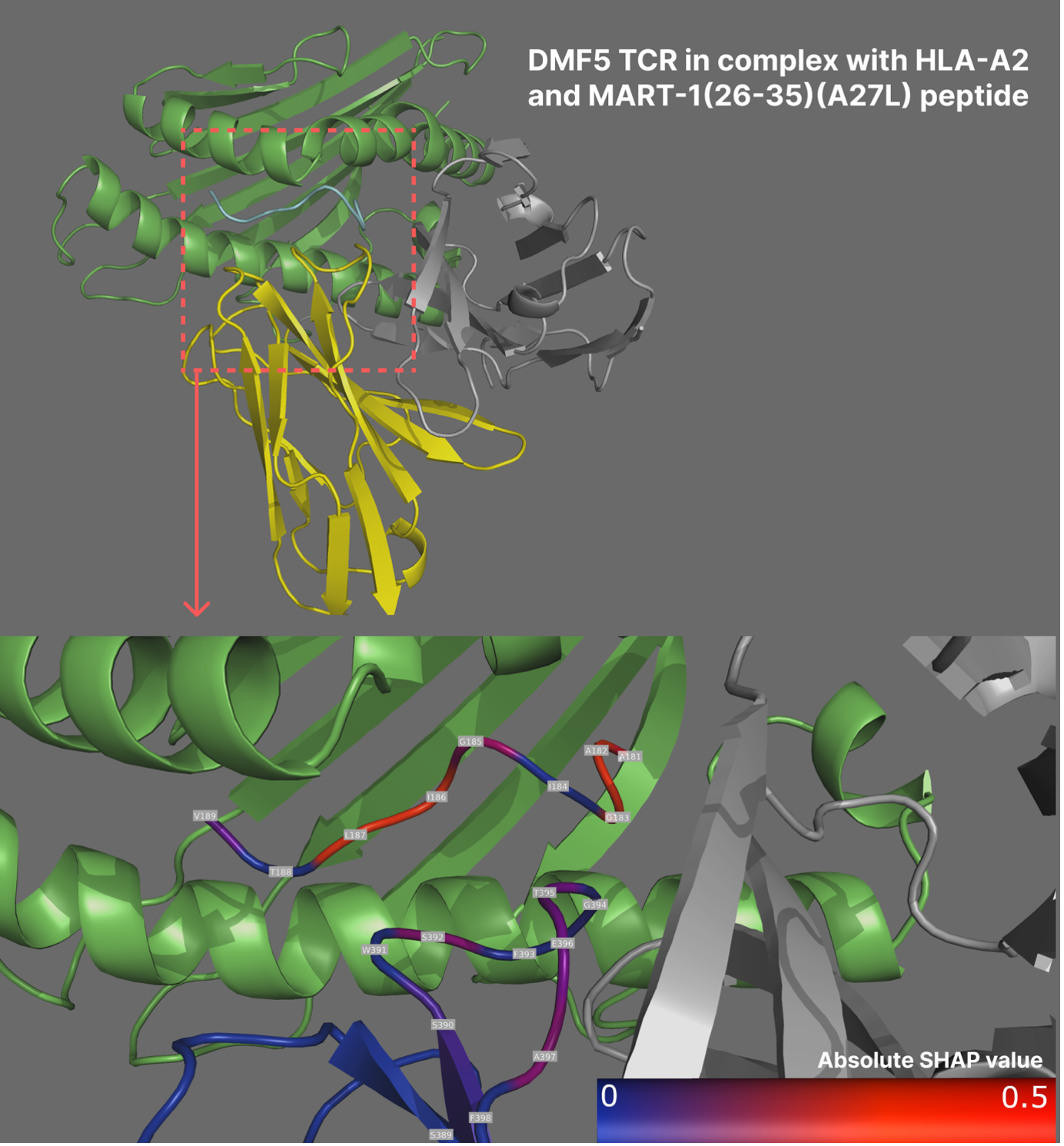

Supplement: S3 Fig — Structure from Hellman et al. 2019 [4]. High SHAP values shown on the known HLA-A2 anchor locations at residue 2 and the C-terminal. Residues 1, 3 and 5–7 have well established preferred and deleterious amino acids, resulting in high absolute SHAP values. Residues at 3 and 5–7 are in position for importance with TCR binding. CDR3-beta residue importance is focused on regions at the binding interface, with low values at the tails. (TIFF) [file pcbi.1012511.s003.tiff]

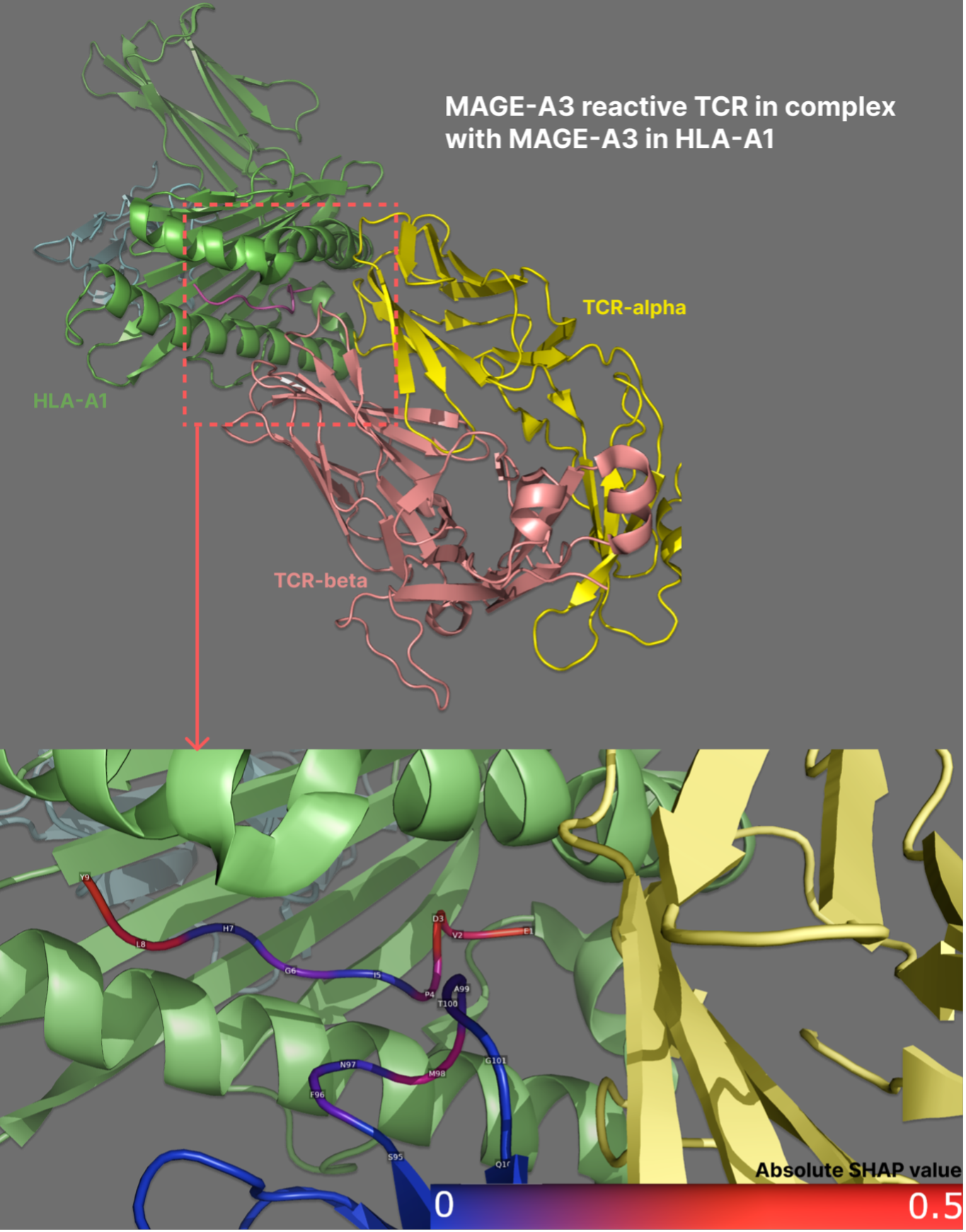

Supplement: S4 Fig — 2016 [5]. HLA-A1 has a very narrow binding motif, especially at positions 2 and the C-terminal. LY is the most preferred peptide suffix for binding. Core residues on both the peptide and CDR3-beta chain likely to be interacting based on structural distance have above background SHAP values. (TIFF) [file pcbi.1012511.s004.tiff]

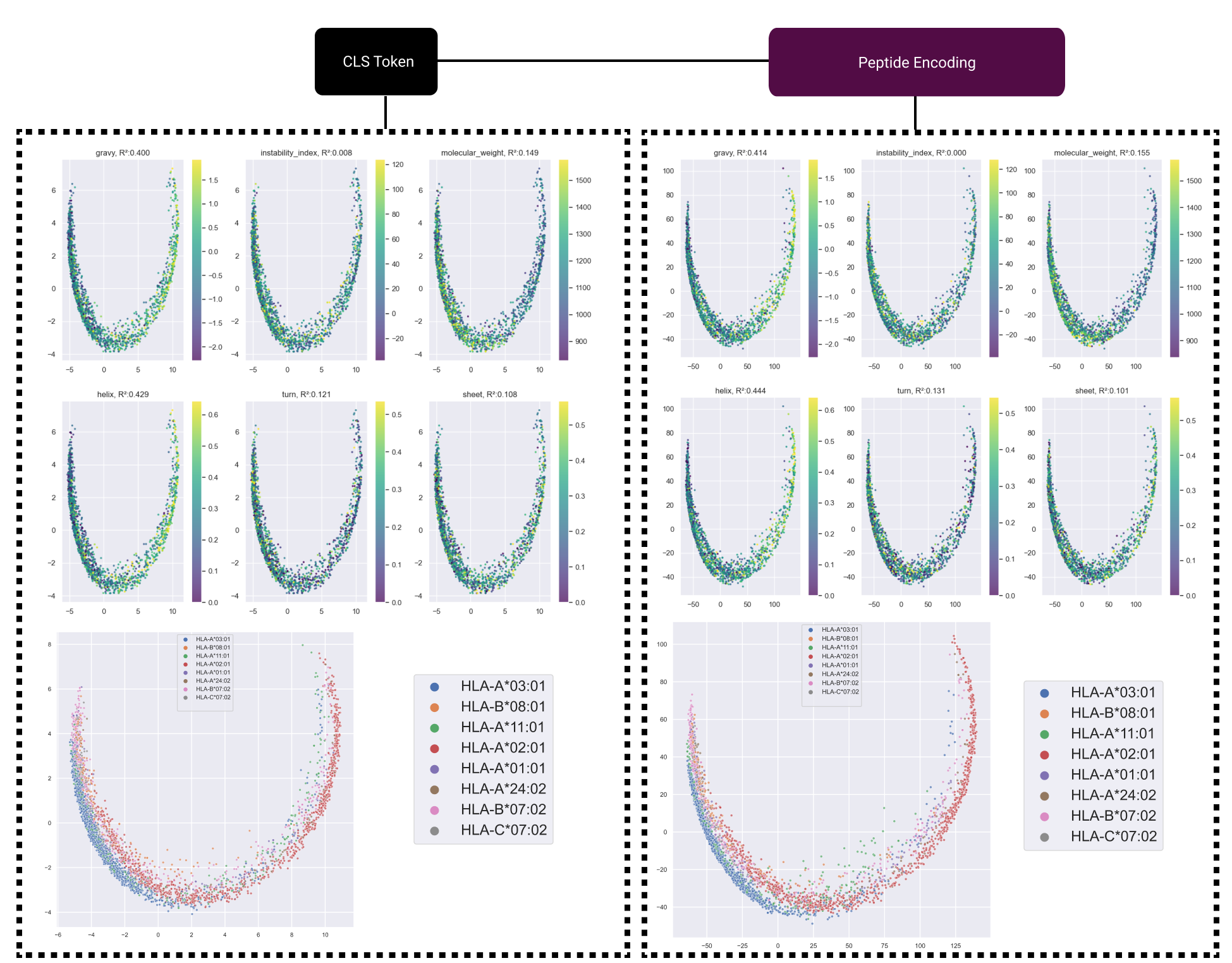

Supplement: S5 Fig — (TIFF) [file pcbi.1012511.s005.tiff]
