# Supplementary material for: A modular protein language modelling approach to immunogenicity prediction
Source: PLoS Comput Biol. 2024 Nov 11;20(11):e1012511. doi: 10.1371/journal.pcbi.1012511 (PMC11581412; doi:10.1371/journal.pcbi.1012511)
Supplement: S1 Text — (DOCX) [file pcbi.1012511.s006.docx]

­­SM 1.1 ESM embeddings as an alternative input to an immunogenicity model

Using foundation models trained on large amounts of data are frequently used as a basis for downstream modelling tasks. The logic is that the foundation model has already learned a high quality, high dimension representation of the input data from which features relevant to the target task can be extracted. This can be particularly useful in low data settings.

Here we replaced the embedding layers of our pMHC immunogenicity model with final layer representations of ESM 2 from FAIR (1). We used the 650M parameter version of ESM 2, which has an embedding size of 1280. This is a substantial increase from our model’s size of 72. To account for this difference the linear layers of the transformer blocks and classification head were had their hidden dimension increased from 256 to 5120 and the number of attention heads increased from 6 to 8. The initial learning rate was decreased from 1e-4 to 1e-5 as at the higher starting rate the training stalled early. Other than these changes the training was performed in the same manner as the main methods.

This version of the model is a large increase in the number of parameters as well as the requirement to run ESM 2 on every input. This increases both the inference runtime of the model and training time. We did not observe any improvement in number of steps to fit the model, with similar training curves seen between both the original version and ESM versions of the model. This could align with results from a recent paper investigating the fine-tuning of ESM for eluted ligand prediction, where the authors describe that the ESM embeddings may not be applicable to the specific task of EL prediction and required fine-tuning end-to-end (2).

Figure S2 shows the performance of the ESM model on the cancer holdout dataset described in Figure 2 of the main paper. The ESM model achieved comparable performance to the version of our model which used a learned embedding layer.

In our design ESM 2 is used as a fixed embedding as the computational resources and time required to fine-tune the entire 33-layer model was beyond the scope of this test. The increased embedding depth and embedded features from ESM 2 did not improve the performance of the model in this setting; however, as more immunogenicity data and structural inputs become available a larger more complex model such as ESM may benefit prediction on this task.

SM 2.1 SHAP importance analysis on structural examples

Figures S3 and S4 show individual examples of the SHAP-based interpretability analysis. Known peptide:HLA:TCR triplets involving MART-1 and MAGE-A3 epitopes were run through the SHAP permutation explainer to identify residues seen as important to the model’s output when changed to values from the background distribution. These SHAP values were then mapped onto structures taken from the protein data bank. The peptide values matched well with the documented binding values for their respective HLAs, documented on IEDB (3). Additional importance was given to residues in the core region found close to residues from the TCR structure. CDR3-beta residues in both cases had raised values in locations near the presented peptide and near zero on the sequence tails.

References

1. Lin Z, Akin H, Rao R, Hie B, Zhu Z, Lu W, et al. Language models of protein sequences at the scale of evolution enable accurate structure prediction [Internet]. bioRxiv; 2022 [cited 2024 Aug 8]. p. 2022.07.20.500902. Available from: https://www.biorxiv.org/content/10.1101/2022.07.20.500902v1

2. Hashemi N, Hao B, Ignatov M, Paschalidis ICh, Vakili P, Vajda S, et al. Improved prediction of MHC-peptide binding using protein language models. Front Bioinform. 2023 Aug 17;3:1207380.

3. Vita R, Mahajan S, Overton JA, Dhanda SK, Martini S, Cantrell JR, et al. The Immune Epitope Database (IEDB): 2018 update. Nucleic Acids Res. 2019 Jan 8;47(D1):D339–43.

4. Hellman LM, Foley KC, Singh NK, Alonso JA, Riley TP, Devlin JR, et al. Improving T Cell Receptor On-Target Specificity via Structure-Guided Design. Molecular Therapy. 2019 Feb 6;27(2):300–13.

5. Raman MCC, Rizkallah PJ, Simmons R, Donnellan Z, Dukes J, Bossi G, et al. Direct molecular mimicry enables off-target cardiovascular toxicity by an enhanced affinity TCR designed for cancer immunotherapy. Sci Rep. 2016 Jan 13;6(1):18851.
