# Supplementary material for: A modular protein language modelling approach to immunogenicity prediction
Source: PLoS Comput Biol. 2024 Nov 11;20(11):e1012511. doi: 10.1371/journal.pcbi.1012511 (PMC11581412; doi:10.1371/journal.pcbi.1012511)
Supplement: Attachment — Submitted filename: Genesis.docx [file pcbi.1012511.s007.docx]

The authors propose Genesis, a modular protein language model for various tasks for neoantigen immunogenicity prediction, which can be potentially helpful in the development of personalized cancer medicine. The existing major challenge in this problem is mainly the limitation of the lack of high-quality data available for training, and the modular methodology authors proposed seems a very convincing approach to help mitigate this problem. Specifically, the authors use encoding modules pretrained from previous prediction tasks or sometimes masked language modeling, and use them as encoders for downstream tasks. Indeed, the authors provide comprehensive experiments on various tasks to show how their models outperform previous baselines. They also provide some visualization and interpretability of their results.

The manuscript is well written and nicely structured. The introduction part is very comprehensive and helps the readers to understand the previous practice in the related areas. The authors then introduce their results, and they present an important message to the readers that adding TCR information indeed helps this task. In the methods part, the authors provide a very detailed description of their data preprocessing, and the control of data leakage is very convincing. Overall, I think the paper fulfills its claim within its scope.

My first question is about the possible shortage of using transformers. The transformer is a powerful deep learning architecture and almost the go-to choice in discrete modeling today, but it can cause problems when the data is limited. For example, they might exploit specific bias patterns in the training data which are not informative in real scenarios. For TCR sequences, I think this is very common because tokens in some regions are often not related to its functions directly. I can see that the authors have provided visualization experiments for interpretability, and the strict selection of validation set has also been convincing. But I think a direct discussion about this issue would be helpful.

I am also curious about the use of masked language modeling loss in TCR transformers. Is there an ablation study why this would be helpful? Also, did the authors consider masking strategies other random masking? I also wonder if using pretrained models, like ProtTrans or ESM2, and attach them to a smaller transformer model would have similar results or even better.
