# Supplementary material for: A modular protein language modelling approach to immunogenicity prediction
Source: PLoS Comput Biol. 2024 Nov 11;20(11):e1012511. doi: 10.1371/journal.pcbi.1012511 (PMC11581412; doi:10.1371/journal.pcbi.1012511)
Supplement: Attachment — Submitted filename: ReviewerResponse.docx [file pcbi.1012511.s008.docx]

Reviewer #1: Summary
Genesis is a modular protein language model designed to enhance immunogenicity prediction for CD8+ reactive epitopes. It comprises a peptide-MHC (pMHC) encoding module trained on three key tasks—binding affinity, eluted ligand prediction, and stability—alongside optional T-cell receptor (TCR) encoding modules and context-specific immunogenicity prediction heads. The model's interpretability analysis identifies weaknesses and biases in existing datasets, offering a better understanding of immunogenicity features.

Minor Comments
1. For all figures, please increase the font size of x and y axis labels. They are currently too small and difficult to read.

We’ve remade all the figures with a higher font size on all the charts, thank you for noting this.

2. What masking rate was used during the pretraining phase?

The rate was 13%, we’ve added this to the methods

3. For Figure 5 feature maps, consider including a PCA-based visualization as a supplementary figure, which may provide easier interpretation. In Figure 6 Panel G, the x-axis label is missing for half of the plot.

Thank you for these comments, we’ve fixed Figure 6 and made the supplementary figure as suggested for the PCA-based visualisation.

4. Regarding data leakage control, why did the authors choose to use 8-mer matching? Is there a specific rationale for this choice? Typically, we see controls based on 3, 6, or 9 (3-based) mers. Additionally, have authors tried identity clustering for example?

8-mer matching in amino acid space was selected based on this being the minimum viable epitope for class 1 HLAs in our dataset, ensuring epitopes which were substrings of each other were not separated into different cross validation folds when this would help with prediction. E.g. in the extreme case this controls for cases where a HLA binds to both a 9 and 10mer variant of the same underlying epitope and both have the same immunogenicity label. Single nucleotide variations in short epitopes can have a large impact on immunogenicity so smaller kmer similarity limits were not considered to be a high priority data leakage risk. For example mutations at hotspots such as KRAS-G12 can produce several epitopes that differ by one amino acid but are only known to be immunogenic in the presence of different HLAs.

5. A more comprehensive analysis of position-specific SHAP values would be beneficial. How do these masking changes relate to known TCR contacts or mutations/variants across alleles? Instead of masking, could direct mutation effects be examined?

We have updated the description of the mechanism behind the SHAP analysis. We use their Permutation Explainer, which rather than masking, one amino acid is replaced with a corresponding amino acid in the same location from a background distribution on each cycle. This in effect is identifying the effect of mutations in a given location aggregated across many iterations. We agree using a masking approach would not be as useful for this task. We’ve added some more details to the methods to make this clear.

To the supplementary information we’ve added 2 examples of our SHAP importance for the epitope and CDR3 displayed on the known structures of pMHC:TCR triplets to demonstrate the model’s focus on relevant residues involved in both the binding to their HLA but also in residues available to the TCR.

Suggestions

Note: Suggestions are not formal comments. Authors are not required to address items in the suggestions section, which do not affect the reviewer's decision.

1. Have you considered using a pretrained model such as ESM2 as your backbone? This approach might yield better performance or faster convergence given its inherent knowledge.

Thank you for this suggestion, which was also shared by the other reviewers. This was on our longer-term roadmap but we have performed some experiments now based on this reviewer feedback. We used ESM 2 representations from the model’s final layer as a replacement for the learned embedding layer. The model was then fit as normal and compared to the original model with learned embeddings. We used the 650M parameter version, increasing the embedding dimension of our model from 72 to 1280 to accommodate their representation as direct input. The size of the internal hidden layers and attention heads was also increased to match the ESM format. We found this resulted in a very similar performance on our holdout dataset for the main pMHC immunogenicity prediction task. This result was somewhat surprising as, like the reviewers, we expected some benefits from large the pre-trained embedding. A previous paper (<https://doi.org/10.3389/fbinf.2023.1207380> , model not available to test) which used ESM as the backbone for their pMHC-EL model found they had to fine-tune ESM end-to-end indicating its embedding was not initially useful for the specific pMHC EL problem they were predicting.

A large disadvantage of utilising ESM in this manner, or indeed fine-tuning it, is that it greatly increases the number of parameters, runtime and therefore cost of training the model. Our early tests showed increasing the transformer block depth beyond 4 layers provided no performance increase. Our work indicates that state-of-the-art immunogenicity prediction levels can be reached with a much shallower model than ESM or similar large protein models. As more data becomes available, especially with paired TCR information, larger models such as ESM may start to show benefits.

2. It is increasingly common to see performance improvements by augmenting protein language models with some degree of structural information, especially for antigens, given the importance of structural complementarity.

Thank you for this point. This is something that is of interest to us but due to the extremely low availability of structural data we view language models as the most practical approach currently. There have been some papers looking at improving computation modelling for TCR-peptide interactions (e.g. <https://elifesciences.org/articles/82813>), but authors generally note these are far short of the accuracy needed for practical usage. As more data becomes available, we agree a structural component may start to show clear benefits – e.g. cancer grand challenge MATCHMAKERS, and other ongoing studies aiming to generate large volumes of this type of data. Similarly, the lack of improvement from the ESM embedding, which can be used as the basis for ESM-Fold, inclusion in the model indicates the current structural datasets don’t yet represent the immune reaction we’re predicting well. We’ve added a point to the discussion about the need to consider adding structural information in future.

3. How would an end-to-end trained model that jointly learns pMHC and TCR features compare to the current approach of separate training?

An issue with training an end-to-end model is mainly the low volume of paired pMHC-TCR data to train such a model. Our early experiments found that starting with an untrained model and going straight to pMHC-TCR inputs struggled to fit or was worse at generalising than the stepwise approach. The issue for this task is that the end goal is the point where the least data is available. A potential alternative option would be to use synthetic triplets data to pre-train the model (something similar is performed in models such as STAPLER as one aspect of training), but this would have the disadvantage of the model not being pre-trained on the relevant pre-training tasks from immunopeptidomics, binding affinity and stability assay data, of which there is a reasonable amount. All of these sub-tasks have been shown to be correlates to immunogenicity. We’ve added this point to the discussion.

Reviewer #2: Comment: In 4.2 Peptide MHC Encoding Module, there are 4 encoder layers in the encoder block. Generally protein LMs like ESM [1], ProtTrans [2] and ProteinLM [3] have >= 12 transformer layers to ensure the model can capture enough information.
Increasing the number of layers and hidden size of your model can help with the performance even more.

Thank you for this suggestion, it was also shared by the other authors. We conducted an experiment using ESM 2 and have added it to the supplementary information. Please see the reviewer 1 response for our full answer on this point.

Question: I wonder have you performed any ablation study on the optimal number of layers in encoder? As well as using general purpose protein language model as the protein sequence encoder, then use the extracted embedding to do the immunogenicity prediction?

We’ve performed ablation studies using cross validation on the training sets going up to 8 layers but found performance levelled off at 4 layers and with deeper models the performance was less consistent between other conditions in the parameter sweep. Our analysis indicated that with the low level of data for the final immunogenicity task, there is a risk of over-parameterisation at the final step of model training. We’ve added this point to the methods.

[1] Rives, Alexander, et al. Biological structure and function emerge from scaling unsupervised learning to 250 million protein sequences.
[2] Elnaggar, Ahmed, et al. ProtTrans: Toward Understanding the Language of Life Through Self-Supervised Learning.
[3] Xiao, Yijia, et al, Modeling Protein Using Large-scale Pretrain Language Model.

Reviewer #3: The review is uploaded as an attachment.

The authors propose Genesis, a modular protein language model for various tasks for neoantigen immunogenicity prediction, which can be potentially helpful in the development of personalized cancer medicine. The existing major challenge in this problem is mainly the limitation of the lack of high-quality data available for training, and the modular methodology authors proposed seems a very convincing approach to help mitigate this problem. Specifically, the authors use encoding modules pretrained from previous prediction tasks or sometimes masked language modeling, and use them as encoders for downstream tasks. Indeed, the authors provide comprehensive experiments on various tasks to show how their models outperform previous baselines. They also provide some visualization and interpretability of their results.

The manuscript is well written and nicely structured. The introduction part is very comprehensive and helps the readers to understand the previous practice in the related areas. The authors then introduce their results, and they present an important message to the readers that adding TCR information indeed helps this task. In the methods part, the authors provide a very detailed description of their data preprocessing, and the control of data leakage is very convincing. Overall, I think the paper fulfills its claim within its scope.

My first question is about the possible shortage of using transformers. The transformer is a powerful deep learning architecture and almost the go-to choice in discrete modeling today, but it can cause problems when the data is limited. For example, they might exploit specific bias patterns in the training data which are not informative in real scenarios. For TCR sequences, I think this is very common because tokens in some regions are often not related to its functions directly. I can see that the authors have provided visualization experiments for interpretability, and the strict selection of validation set has also been convincing. But I think a direct discussion about this issue would be helpful.

Thank you for this note about potential issues using transformers for this task. We agree that there is an issue with models learning unwanted features, especially on the TCR tasks where the data is more limited. For the TCR tasks in particular this is why we compared to the STAPLER model using their data split, where removing data leakage was their priority to demonstrate some other published models may have overestimated performance on TCR-epitope specificity in unseen epitopes. We have added some detail about this to the discussion. In particular we think it is important to rerun all of our visualisation experiments for new versions of out model, as adding new data from new sources could have an unwanted impact due to differences in the underlying distributions in the data.

I am also curious about the use of masked language modeling loss in TCR transformers. Is there an ablation study why this would be helpful? Also, did the authors consider masking strategies other random masking? I also wonder if using pretrained models, like ProtTrans or ESM2, and attach them to a smaller transformer model would have similar results or even better.

Thank you for these comments. We found that without pre-training the TCR encoding branch with the masked language modelling we were unable to fit the model well, so no specific ablation study results were reportable for comparison. This is likely due to the low volume of TCR data with known reactivities to train with. For this reason, some form of pre-training has become common in these sorts of models.

Your point about using models, such as ESM, as the basis for our model was very welcome and shared by the other reviewers. We ran an experiment using ESM 2 final-layer representations as input the model, now in the supplementary information. Please see the response to reviewer 1 for a longer form answer to this point.
